# Prevalence of Low Serum Alkaline Phosphatase and Hypophosphatasia in Adult Patients with Atypical Femur Fractures

**DOI:** 10.1007/s00223-022-00949-1

**Published:** 2022-02-28

**Authors:** Eleni Tsiantouli, Emmanuel Biver, Thierry Chevalley, Robert Petrovic, Didier Hannouche, Serge Ferrari

**Affiliations:** 1 Division of Bone Diseases, Geneva University Hospitals and Faculty of Medicine, University of Geneva, Geneva, Switzerland; 2 Division of Orthopedic Surgery, Department of Surgery, Geneva University Hospitals and Faculty of Medicine, University of Geneva, Geneva, Switzerland; 3 Institute of Medical Biology, Genetics and Clinical Genetics, University Hospital Bratislava, Bratislava, Slovakia; 4 Service and Laboratory of Bone Diseases, Department of Medicine, Geneva University Hospitals (HUG), 64 avenue de la Roseraie, 1205 Geneva, Switzerland

**Keywords:** Atypical Femur Fracture, Hypophosphatasia, Alkaline Phosphatase, Rare bone disease, Bisphosphonates, Osteoporosis

## Abstract

Hypophosphatasia (HPP) is a rare genetic disorder characterized by low serum alkaline phosphatase (ALP), its manifestations may include atypical femoral fractures (AFF). However, the prevalence of low serum ALP and HPP in patients with AFF remains unknown. We retrospectively analyzed ALP levels and clinical manifestations compatible with HPP in 72 adult patients with confirmed AFF by chart review. ALP values were compared with those of a control group of patients with prior proximal femoral fracture during antiresorptive treatment (*n* = 20). Among the AFF patients, 18 (25%) had at least one serum ALP value ≤ 40 IU/L, although in all but one case, at least one ALP value > 40 IU/L was also detected at another time point. Most low ALP values were associated with antiresorptive treatment (*P* = 0.049) and lowest levels of ALP did not differ between the AFF and the control groups (*P* = 0.129). However, low ALP values among AFF patients were associated with a higher rate of bilateral AFF (50% vs 22%, *P* = 0.025), metatarsal fracture (33% vs 7%, *P* = 0.006), and with trends for more frequent use of glucocorticoid (22% vs 8%, *P* = 0.089) and proton pump inhibitor (61% vs 44%, *P* = 0.220). In one AFF patient with low ALP and clinical suspicion of HPP, a rare pathogenic heterozygous variant of the *ALPL* gene was identified. In conclusion, low ALP values are common among subjects with AFF and mainly related to concomitant antiresorptive medication. Hence, low serum ALP has low specificity for HPP among AFF patients.

## Introduction

Atypical femoral fractures (AFFs) are uncommon and have been associated mainly with prolonged use of bisphosphonates and denosumab, although about 10% occur in the absence of exposure to antiresorptive treatment [1–4]. The pathophysiological mechanisms of AFF remain poorly understood, but some clinical, biomechanical, and genetic factors have been implicated in the risk of AFF [5, 6]. Among those, recent studies report an increased risk for subtrochanteric and diaphyseal femoral fractures in patients with hypophosphatasia (HPP) [1, 6].

HPP is an inborn error of metabolism characterized by a quantitative deficit of alkaline phosphatase (ALP), due to a loss‐of‐function mutation in the *ALPL* gene (also known as *TNSALP* gene) that encodes the tissue non-specific ALP [7]. The adult form of HPP presents with a wide range of clinical manifestations, including skeletal alterations due to defective bone mineralization such as osteopenia, premature tooth loss, rickets, osteomalacia, delayed fracture healing, stress fractures of metatarsal bones, AFF, or cortical stress fractures (pseudo-fractures) which can resemble bisphosphonate‐associated incomplete AFF when localized to the lateral side of the femoral shaft [8]*.* In addition, HPP manifests with clinical signs and non-specific and mild extra-skeletal symptoms such as seizures, nephrocalcinosis, kidney stones, muscle weakness, chronic pain, and fatigue often overlapping with other metabolic bone diseases. For this reason, many cases of adult HPP remain un- or misdiagnosed, and, subsequently, are treated wrongly or even remain untreated.

Hence, the occurrence of an AFF in adulthood should raise clinical suspicion of an undiagnosed mild form of HPP, particularly when associated with other biochemical and clinical signs consistent with the disease, which should then be confirmed by genetic sequencing of *ALPL* gene. However, the sensitivity of genetic screening for HPP may be impaired by the lack of patient selection based on the probability of bearing HPP mutations, i.e., on the presence or absence of persistently low serum ALP values [9, 10].

The aim of our study was to investigate the prevalence of low serum ALP values in a cohort of subjects with AFF and compared to a control group of patients with common proximal femur fractures receiving bisphosphonates (BPs). As a secondary outcome, we also explored the influence of antiresorptive medications on ALP values, since these drugs are a major risk factor of AFF. Eventually, TNSFALP gene mutations were screened in AFF patients who presented with persistently low ALP values and clinical manifestations typical of the disease.

## Methods

### Study Design and Patients

This was a retrospective observational chart review study. The primary endpoint was the prevalence of low ALP values in patients with AFF. The secondary endpoint of the study was to compare serum levels of ALP of patients with AFF with a control group of patients who presented with prior hip fragility fracture during antiresorptive treatment and to evaluate the influence of antiresorptive treatment on ALP levels.

In the AFF cohort 72 patients aged 18 years or older, who were diagnosed with AFF and hospitalized at the Service of Orthopedics at the Geneva University Hospitals between 2000 and 2020 were included. The presence of an AFF was defined according to the criteria of the American Society for Bone and Mineral Research [1]. Exclusion criteria included rare bone disease, current bone, primary liver or metastatic cancer, liver transplantation, autoimmune liver disease, and lack of ALP data. In the control group, adult patients hospitalized with a hip fracture at the Service of Orthopedics at the Geneva University Hospitals between 2007 and 2017 and who had previously been exposed to BPs were included. Three hundred patients with proximal femur fractures were screened for concomitant BP use and only 23 patients were identified as having received antiresorptive treatment at the time of fracture (control group). Of those, 3 patients with current bone or gastrointestinal cancer, previous liver transplantation, and rare bone disease were further excluded.

Clinical and biochemical data were collected before, during, and after hospitalization from medical records of the hospitals and from treating physicians. Previous antiresorptive treatment included oral (alendronate, ibandronate, risedronate, pamidronate) or intravenous (zoledronic acid) bisphosphonates as well as subcutaneous denosumab. Oral vitamin D supplements could be administered alone or together with calcium in doses of 800 UI–1200 UI/day. Additionally, the use of corticosteroids, which can reduce ALP activity and increase the risk of hip fracture and AFF [11], were evaluated. Corticosteroid use included prednisone administration at a range dose 2–30 mg/day. PPIs, including esomeprazole, omeprazole, pantoprazole in standard doses used at least for 1 year, were also evaluated since preclinical studies suggested that IPPs may decrease ALP expression [12] and that IPPs use has been associated with AFF. The study was approved by the Ethics committee Geneva, Switzerland.

### Assessments

Clinical data assessed of all included patient demographics, medical history, previous drug treatments, and radiographs. Anamnestic data such as chronic fatigue and chronic pain syndrome, nephrolithiasis, and diagnosis of dementia or other neuropsychiatric diseases were collected during hospitalization or ambulatory visits. Evaluation of osteoporosis was performed using bone densitometry (dual-energy X-ray absorptiometry, DXA) using the available data before and after fracture. Medical records did not include dental history of the patients. At least one serum ALP measurement was prospectively evaluated from every patient. Fractures were diagnosed by conventional radiographs and evaluated by at least one senior consultant of the Service of Orthopedics and of the Division of Bone Diseases at the Geneva University Hospitals.

### ALP Measurement

Quantitative determination of ALP in human serum and plasma was performed until 2015 by Beckman Coulter System and after 2015 by a standardized colorimetric assay using a Roche/Hitachi automated clinical chemistry analyzer (Roche Diagnostics, Mannheim, Germany). In short in Roche/Hitachi, p-nitrophenyl phosphate is hydrolyzed by phosphatases in the presence of magnesium and zinc ions to form phosphate and p-nitrophenol. The p-nitrophenol released is proportional to ALP activity and can be measured photometrically. Precision was determined using human samples and controls in an internal protocol. The lower detection limit of ALP was 5 IU/L, the normal range in adults was 30–125 IU/L. However, the cut-off for low ALP was defined as 40 IU/L in our study according to the age-adjusted range of the Canadian Laboratory Initiative in Pediatric Reference Intervals (CALIPER) [13–15].

### Vitamin D and Vitamin B6 Measurements

Quantitative determination of vitamin D (25-OH vitamin D) in human serum was performed by COBAS 8000ECLIA (Roche Diagnostics, Rotkreuz, Switzerland). In an adult population, values less than 25 nmol/L indicate vitamin D deficiency, values between 25 and 50 nmol/L vitamin D insufficiency and levels over 75 nmol/L reflect optimal supply.

For the determination of vitamin B6 (pyridoxal 5′-phosphate, PLP) levels, high-performance liquid chromatography (HPLC)-fluorescence methods were used, the reference range being 12–75 nmol/L.

### Molecular Genetic Analysis of the ALPL Gene

All exons and flanking parts of introns (20 bp) of ALPL gene by direct sequencing and compared with refseq: NM_000478.5; promoter and deep intron regions were not analyzed. Possible deletions and duplications (copy number variants—CNV) of ALPL gene were analyzed by MLPA (kit P484-ALPL, lot: A1-0918, MRC Holland, Amsterdam, the Netherlands). These analyses were carried out by Robert Petrovic at the University Hospital of Bratislava.

### Statistics

Data are presented as medians with interquartile ranges or percentage. Comparisons between groups were performed using non-parametric Mann–Whitney tests for continuous data and *χ*^2^ tests for categorical variables. *P* values < 0.05 were considered to indicate significant statistical differences between groups. All analyses were performed using STATA software, version 14.0 (StataCorp LP).

## Results

### Patient Characteristics

The study population included 72 patients (65 females, 7 males) with AFF and at least one available ALP value (Table 1). Among them, 10 patients had not been treated with antiresorptive treatment (AR) while 62 patients were treated with AR, with a duration of AR [median (interquartile range)] of 8 (6) years. These two subgroups were not different concerning age, sex, proportion of bilateral and simultaneous AFF, metatarsal fractures, prevalence of osteoporosis, and corticosteroids or PPIs use. Subjects without AR treatment had a higher body mass index (BMI) (*P* = 0.017). Compared with the AFF group treated with AR, patients in the control group, i.e., with hip fractures on BPs therapy, were slightly older (*P* = 0.026), had lower BMI (*P* = 0.011), a trend for shorter AR duration (*P* = 0.071), and higher CTX levels (*P* = 0.002).

**Table 1:** Patient characteristics according to the use of antiresorptive drugs

	AFF group						*P* value^a^	Hip fracture group		*P* value^b^
	Total *n *= 72		No antiresorptive drug use *n* = 10		Antiresorptive drug users *n* = 62			Antiresorptive drug users *n* = 20		
	*n*	Median (IQR) or %	*n*	Median (IQR) or %	*n*	Median (IQR) or %		*n*	Median (IQR) or %	
Age (years)	72	77 (16)	10	77 (22)	62	77 (15)	0.775	20	82 (13)	**0.026**
Sex (female, %)	72	90%	10	90%	62	90%	0.975	20	90%	0.966
BMI (kg/m^2^)	66	24.3 (5.8)	8	29.2 (7.4)	58	24.0 (5.4)	**0.017**	16	21.2 (3.0)	**0.011**
Fractures characteristics										
Bilateral AFF (%)	72	29%	10	10%	62	32%	0.151	NA	NA	NA
Bilateral simultaneous AFF (%)	21	76%	1	100%	20	75%	0.567	NA	NA	NA
Metatarsal fracture (%)	72	14%	10	0%	62	16%	0.171	NA	NA	NA
Pertrochanteric fracture, (%)	NA	NA	NA	NA	NA	NA	NA	NA	13 (65%)	NA
Femoral neck fracture, (%)	NA	NA	NA	NA	NA	NA	NA	NA	7 (35%)	NA
Lowest ALP values (IU/L)	72	58 (46)	10	86 (40)	62	56 (39)	**0.040**	20	53 (16)	0.313
ALP ≤ 40 IU/L	72	25%	10	0%	62	29%	**0.049**	20	20%	0.428
Patients on AR therapy, (%)	72	86%	10	0%	62	100%	** < 0.001**	20	100%	NA
Type of AR therapy	72	A, I, Z, D, P	10	0	62	A, I, Z, D, P	NA	20	A, I, Z, D	NA
Duration of AR (years)	70	6 (8)	10	0 (0)	60	8 (6)	** < 0.001**	19	4 (7)	0.071
Osteoporosis (%)^c^	56	45%	6	17%	50	48%	0.145	NA	NA	NA
Corticosteroid users (%)^d^	71	11%	10	10%	61	11%	0.891	NA	NA	NA
PPI users (%)	72	49%	10	50%	62	48%	0.925	NA	NA	NA
25-OH Vitamin D (nmol/L)	45	60 (33)	2	58 (100)		60 (33)	0.956	0	NA	NA
CTX (ng/mL)	37	273 (224)	1	1058 (0)	36	270 (216)	0.111	13	442 (250)	**0.002**

*A* alendronate, *AFF* atypical femur fracture, *BMI* body mass index, *ALP* alkaline phosphatase, *D* denosumab, *I* ibandronate, *P* pamidronate, *PPI* proton pump inhibitors, *NA* not applicable

^a^AFF with versus AFF without antiresorptive drug use

^b^Hip fracture group vs AFF with antiresorptive drug use

^c^Osteoporosis defined as at least one T-score ≤ –2.5 SD at the lumbar spine, total hip, or femoral neck

^d^Range of prednisone dose, 2–30 mg/day

*P*-values in bold < 0.05 are considered statistically significant

### ALP Values According to Fracture Type

In order to more precisely address the prevalence of low ALP values in AFF patients, we specifically analyzed the lowest of multiple ALP values assessed. In one patient with alcoholic cirrhosis, four ALP measurements were above the upper limit of normal due to acute liver dysfunction and therefore excluded from analysis.

The median of lowest ALP value was 53 IU/L (range 24–86 IU/L) in the control group, hence not different from all AFF patients (58 IU/L, range 13–322 IU/L, *P* = 0.129) and AFF patients with AR (56 IU/L, range 13–322 IU/L, *P* = 0.313). The proportion of patients with low ALP values (≤ 40 IU/L) was also not different between groups (AFF vs control, 25% vs 20%, *P* = 0.643; AFF with AR vs control, 29% vs 20%, *P* = 0.428) (Table 1).

### Characteristics of AFF Patients According to ALP Values

**Fig. 1:**
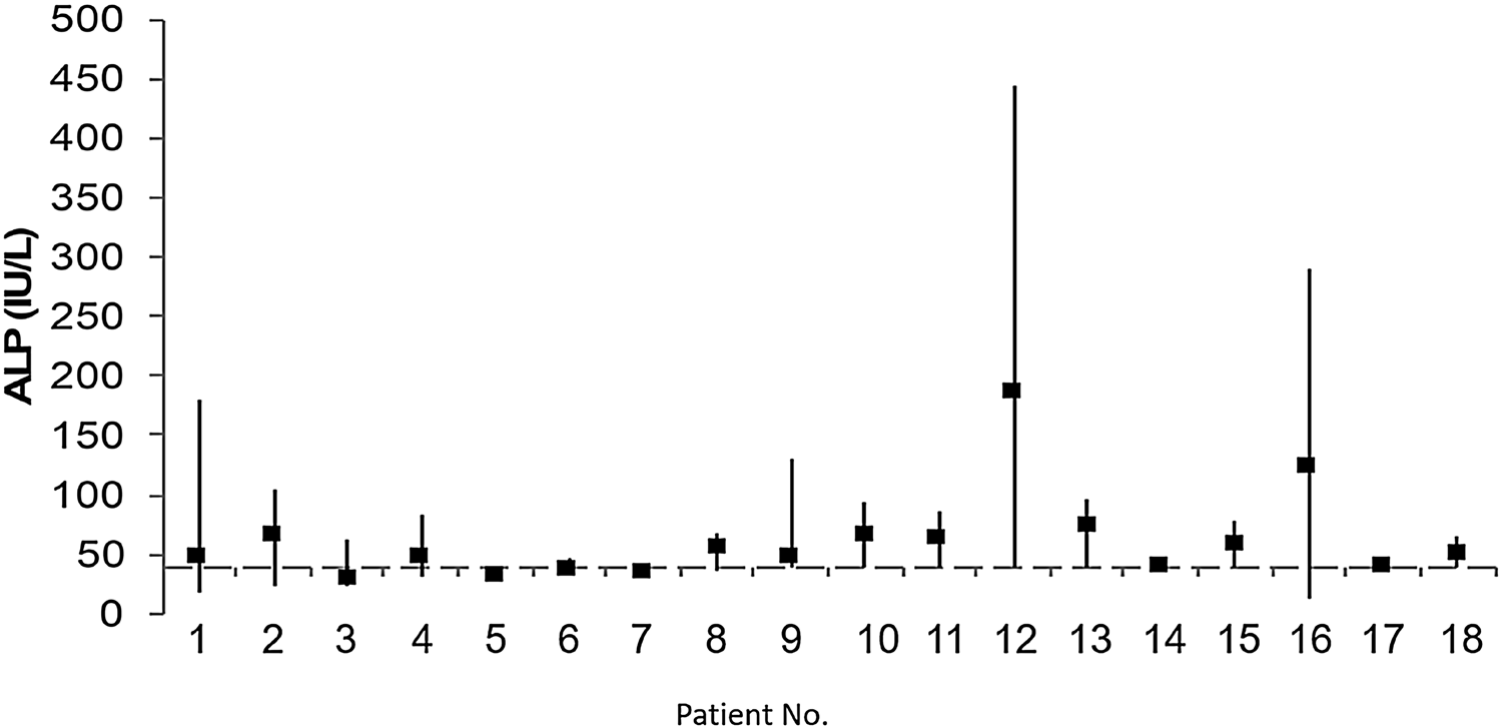
Mean and range value of serum ALP in AFF patients with at least one ALP value ≤ 40 IU/L; ALP: alkaline phosphatase

Fifty-four patients did not have any ALP values ≤ 40 IU/L (median lowest ALP 72 IU/L, range 42–322 IU/L), while 18 AFF patients had at least one ALP value ≤ 40 IU/L (median lowest ALP 38 IU/L, range 13–40 IU/L) (Table 2 and Fig. 1). AFF patients with ALP values ≤ 40 IU/L did not differ from those with values > 40 IU/L for most clinical characteristics, including age, sex, BMI, prevalence of osteoporosis, and duration of AR therapy. However, those with ALP concentrations ≤ 40 IU/L had all been exposed to AR (100% vs 81%, *P* = 0.049), among whom 17 had received BPs and one denosumab for a median of 8 years. They also experienced more bilateral AFF (50% vs 22%, *P* = 0.025), which were simultaneous in 67% and more metatarsal fractures (33% vs 7%, *P* = 0.006), and they were more likely to use glucocorticoids (22% vs 8%, *P* = 0.089). To note that among those, atypical femur fractures healed without complications in 13 patients and resulted in the development of pseudoarthrosis in four patients.

**Table 2:** Characteristics of patients with AFF according to alkaline phosphatase values

	ALP ≤ 40 IU/L *n* = 18		ALP > 40 IU/L *n* = 54		*P* value
	*n*	Median (IQR) or %	*n*	Median (IQR) or %	
Age (years)	18	76 (15)	54	78 (16)	0.639
Sex (female, %)	18	89%	54	91%	0.818
BMI (kg/m^2^)	17	25.2 (5.3)	49	23.8 (5.2)	0.135
Fractures characteristics:					
Bilateral AFF (%)	18	50%	54	22%	**0.025**
Bilateral simultaneous AFF (%)	9	67%	12	83%	0.375
Metatarsal fracture (%)	18	33%	54	7%	**0.006**
Lowest ALP values (IU/L)	18	38 (9)	54	72 (36)	** < 0.001**
Patients on AR therapy, (%)	18	100%	54	81%	**0.049**
Type of AR therapy	18	A, I, Z, D	54	A, I, Z, D, P	NA
Duration of AR (years)	18	8 (7)	52	5 (8)	0.198
Osteoporosis (%)^a^	14	29%	42	50%	0.162
Corticosteroid users (%)^b^	18	22%	53	8%	0.089
PPI users (%)	18	61%	54	44%	0.220
25-OH Vitamin D (nmol/l)	12	43 (39)	33	63 (25)	0.390
CTX (ng/ml)	9	275 (95)	28	270 (316)	0.944

*AFF* atypical femur fracture, *BMI* body mass index, *A* alendronate, *D* denosumab, *I* ibandronate, *P* pamidronate, *PPI* proton pump inhibitors, *Z* zoledronate, *NA* not applicable

^a^Osteoporosis defined as at least one T-score ≤ –2.5 SD at the lumbar spine, total hip, or femoral neck

^b^Range of prednisone dose, 2–30 mg/day

*P*-values in bold < 0.05 are considered statistically significant

### ALP Values According to Antiresorptive Drugs Use in Patients with AFF

**Fig. 2:**
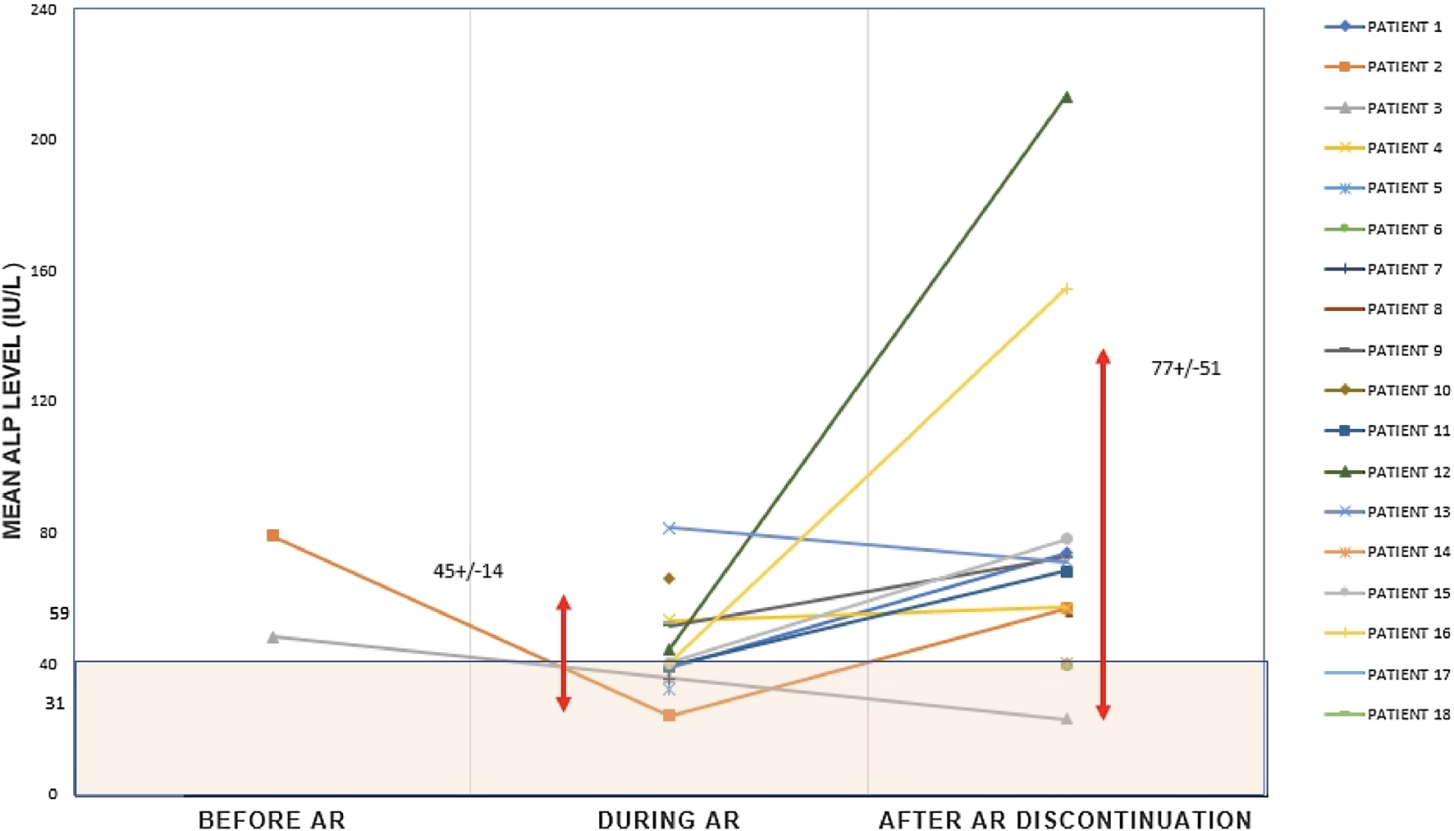
ALP values in AFF patients with at least one ALP value ≤ 40 IU/L before, during, and after discontinuation of antiresorptive treatment; mean values during and after AR treatment ± SD; ALP: alkaline phosphatase; AR: antiresorptive, mean values during and after antiresorptive treatment ± SD

Among patients with AFF, ALP values were lower in AR users compared with AR non-users (56 IU/L vs 86 IU/L, *P* = 0.040). The proportion of patients with low ALP values (≤ 40 IU/L) was also significantly higher in AR users compared with non-users (29% vs 0%, respectively, *P* = 0.049) (Table 1). In addition, in most of low ALP patients with AFF, ALP concentrations exceeding 40 IU/L were also measured at some point of time (Fig. 1), particularly off antiresorptive therapy (Fig. 2), indicating that AR, rather than HPP, was the main reason for their low serum ALP values.

### HPP in AFF Patients with Low ALP

Among the 18 AFF patients with at least one ALP ≤ 40 IU/L, seven patients were considered to have low ALP values in more than one measurement during or after discontinuation of antiresorptive treatment (range 13–40 IU/L). These patients are reported in more detail in Table 3. Vitamin B6 (pyridoxal 5-Phosphate, PLP) level was available only in 2 of these subjects and was not elevated. A number of non-specific clinical signs and symptoms compatible with HPP were found in these patients, including chronic fatigue and primary pain in three patients and gait disorders in one female patient. Two of the seven patients were diagnosed with nephrolithiasis, whereas only one had a metatarsal fracture. Four patients presented with neuropsychiatric disorders, and one patient was suffering from severe heart disease. Three out of seven patients with repeatedly low ALP value**s** were alive at the time of evaluation and consented to provide blood samples for *ALPL* DNA sequencing. In one patient, a rare pathogenic heterozygous variant (c.787T > C, p.Tyr263His) was identified which is associated with a likely benign form of HPP [16]. In the other two patients no HPP mutation was detected.

**Table 3:** AFF patients with at least one ALP ≤ 40 IU/L during and after discontinuation of antiresorptive treatment

Pat. no	Sex	Age	Clinical symptoms	Median ALP (IU/L)	Vitamin B6 (nmol/L)^a^	Bisphosphonate(s) used	ALP range (IU/L)
1	F	67	Chronic primary pain, osteoporosis, chronic fatigue	47	NA	Ibandronate	19–181
2	F	85	Osteoporosis with vertebral fractures, nephrolithiasis	53	NA	Alendronate, ibandronate	24–104
3	F	58	Severe osteoporosis, metacarpal fractures, mobility impairment, multiple vertebral fractures	31	13	Alendronate	31–62
15	F	77	Chronic primary pain, osteoporosis, chronic fatigue, wrist fracture, vertebral fracture, repetitive falls	40	22	Zoledronate	14–40
6	F	68	Osteoporosis, chronic primary pain	36.5	NA	Alendronate	34–48
17	F	90	Osteoporosis, vertebral fractures	39	NA	Ibandronate	38–44
7	F	75	Osteoporosis, metatarsal, and vertebral fractures nephrolithiasis	35	NA	Alendronate, risedronate, ibandronate	35

*ALP* alkaline phosphatase, *F* female, *M* male, *NA* not assessed

^a^Vitamin B6 normal range 12–75 nmol/L

## Discussion

Our results show that low ALP values are a common finding in patients with AFF and that these values are mainly associated with concurrent antiresorptive medication, similar to BPs-treated subjects with typical hip fractures.

In almost a quarter of AFF patients, at least one low ALP value ≤ 40 IU/L was detected during the observation period. However, ALP values varied greatly and in all patients except one, higher ALP values (> 40 IU/L) were also measured at some time point. Literature data on the prevalence of low ALP levels in AFF patients are scarce and existing data are mostly related to bisphosphonate treatment. In a previous small study, low ALP levels (< 55 IU/L) were observed in five of ten (50%) long-term bisphosphonate-treated patients with AFF and in five out of 13 (38%) bisphosphonate-treated control patients who did not experience any complication [17]. In another study, ALP concentrations were within the reference range in all eight bisphosphonate-treated AFF patients studied [18]. There are no data on serum ALP concentrations in AFF patients in a hospital setting.

In our study 100% of patients with at least one ALP value ≤ 40 IU/L had a history of long-term antiresorptive treatment, mainly with bisphosphonates. These results confirm the well-known association of low ALP values with long-term bisphosphonate treatment [19]. Bisphosphonates reduce bone ALP by about 40% [19, 20] in response to suppression of osteoclast activity, even in patients presenting with excessive ALP levels such as in Paget’s disease [21]. Dose-dependent inhibition of ALP activity by alendronate, pamidronate, and zoledronate by 42–93% was also observed in vitro [22]. Thus, the low ALP values as detected in most of our AFF patients most likely resulted from antiresorptive treatment with bisphosphonates.

However, not all of our patients on antiresorptives show low ALP values, which would suggest that the concurrence of low ALP and antiresorptive treatment may increase the patients’ vulnerability for experiencing AFF, in addition to other risk factors of AFFs such as ethnical group, pretreatment BMD, glucocorticoid use, weight, and height [4]. On the other hand it can be speculated that consistently low ALP values during antiresorptive treatment reflect better adherence and higher exposure to pharmacological treatment. This may then result in an increased risk of experiencing AFF. For this reason, special attention in terms of AFF risk should be paid in the care of patients who present with persistently low serum ALP values while receiving long-term antiresorptive treatment, since the latter has been associated with an absolute risk of up to 100 AFF per 100,000 person-years [1, 3, 4, 23].

When analyzing specific subgroups of patients, bilateral AFF more frequently occurred in patients with at least one ALP value ≤ 40 IU/L than in patients whose ALP levels were > 40 IU/L. This finding would suggest that low ALP may increase the risk for bilateral AFF. Furthermore, the proportion of patients treated with corticosteroids or PPIs among AFF patients tended to be higher in the low ALP group compared with the ALP > 40 IU/L group. This finding may suggest a contribution of PPIs and corticosteroids on bone metabolism in our AFF patients. Corticosteroid treatment has been shown to mainly decrease ALP levels [24, 25], whereas the effect of PPI administration on ALP levels is less clear [26–29]. An increased risk for fractures has been reported in both patients treated with PPIs and corticosteroids [12, 24–26, 30].

When specifically looking for signs and symptoms compatible with HPP in AFF patients with at least one ALP ≤ 40 IU/L during or after discontinuation of antiresorptive treatment , we identified seven patients in whom chronic fatigue, primary pain, nephrolithiasis, neuropsychiatric disorders, and severe heart disease occurred. Out of three patients who finally had genetic testing, a pathogenic heterozygous variant associated with HPP [16] could eventually be detected in one patient. Since we could not perform genetic testing in all low ALP patients with AFF, we are not able to calculate the actual prevalence of HPP in our cohort. However, HPP appears to be uncommon in our setting. Studies on a possible association between AFF and HPP show contradicting results. On one hand a genome-wide association and candidate gene study failed to identify common genetic variants of AFF consistent with HPP (*n* = 51) [9]. On the other hand, another study showed that AFF is common in HPP patients with 10% of patients with confirmed HPP (*n* = 150) having had at least one AFF [10]. Hence, although AFF is recognized as a common complication of HPP, the prevalence of HPP is rather low in the population of AFF patients receiving antiresorptive treatment. Antiresorptive treatment is not recommended in HPP patients.

There are some limitations of this study that can affect the interpretation of the results. First, the design is retrospective, which may have increased the risk for selection bias. This is, however, unlikely since during the whole observation period virtually all AFF cases were referred by orthopedic surgeons to the bone diseases team for consultation. Also due to the retrospective nature of this study, we were not able to perform genetic testing in all patients of our cohort with clinical suspicion of HPP. Second, HPP clinical signs and symptoms used to screen for HPP are non-specific and data were missing for some of them such as dental anomalies as well as vitamin B6 and vitamin D measurements that were only available in some participants. Therefore, we cannot exclude having missed a patient with clinical and/or biochemical suspicion of HPP in our cohort. Third, the number of controls, i.e., the group of patients having typical hip fractures with antiresorptive treatment is low. Strengths of this study are the large number of AFF patients included with a long follow-up period and the inclusion of an appropriate control group.

In conclusion, among patients with AFF, the prevalence of low ALP values < 40 IU/L is quite common (22.5%). However, low ALP values are most commonly associated with antiresorptive treatment and values measured prior and/or after this treatment generally are within the normal range. Hence, from a clinical perspective, ALP values in patients with AFF should be reappraised at a later time point, i.e., when the fractures are healed and the antiresorptive withdrawn, in order to avoid excessive genetic screening for HPP.
